# Lesions in a Case of Immobilite

**Published:** 1883-04

**Authors:** A. W. Clement

**Affiliations:** Student Montrel Veterinry College


### CASE DEPARTMENT.

### I.—LESIONS IN A CASE OF IMMOBILITÉ.

BY A. W. CLEMENT,

*Student Montrel Veterinry College.*

The subject was a bay gelding, about eighteen years of age, belonging to a cartage company in this city. About four years ago he was said by the men to have had a "touch of the sun,"—since then he had performed his ordinary work until last spring, when he presented symptoms of "Immobilité." He soon recovered and was again put to work. About six weeks ago he had another attack and was useless for work afterward. He exhibited a great disinclination to move, especially to work, and would frequently paw the air with his front feet. He was finally brought to the college and destroyed for the purpose of making a post mortem examination.

All the organs were found normal with the exception of the spleen, which was somewhat hypertrophied, and the brain, which presented the following conditions:

Nothing special about the dura or pià-mater, the canals of the latter were moderately injected. The organ was firm and hard, the substance presented no special alteration, the lateral ventricles were not distended with fluid but appeared of about the natural size, the lining membrane was clear, not granular. Attached to each choroid plexus was a tumorous-like mass about the size of a bean, which was grayish yellow in color, with a rough, irregular surface, tolerably firm. On sections it was gritty in spots, the cut surface presented numerous small rounded bodies about the size of the head of a pin; these could be easily turned out and had a glistening somewhat crystalline appearance. On examination these were found to be made up of masses of cholesterine plates closely aggregated together and mixed with fatty and calcarious matter; these were mixed with brown hæmatoiden grains. There were a few concentricaly arranged bodies, but not sufficient to cause a special feature in the growth. A fibrous stroma pervades the tumors which present spots of fatty and pigmentary degeneration. The velum-interpositum was intimately united with the fornix and surface of the thalami, particularly the left thalamus which was torn in the removal. The petuitary body was about double the normal size, firm, and on sections had a grayish brown color, the anterior portion was more gray than the posterior and there was a distinct line of separation between them. There was a small hæmorrhage in the central portion. The base of brain immediately behind the optic chiasm presented a distinct depression produced by the enlarged petuitary gland, sections of the gland showed it to be composed of cells most of which were rounded in shape and about the size of a colorless blood corpuscle.

On hardening the gland and cutting sections the alveolar structure of interior portions was well seen, and the organ appeared to be in a condition of simple hypertrophy.

The points of interest in the case is the condition of hypertrophy of the petuitary body which has not often been noted in this connection, The tumors of choroid plexure are frequently found. There was no dropsy of ventricles which is present in many instances and is thought by some to be the cause of the disease. Indeed, the lesions in this affection are so diverse that we cannot speak of any one as constant and characteristic, but they would appear to have this effect in common, viz., an increase in the intracranial pressure.

[Tumors similar to those mentioned in connection with the choroid plexuses are quite frequently met with, both in human and comparative pathology. We should be inclined to class them as angiolithic sarcoma. Whether they give rise to any symptoms during life is still an open question.—EDS.]

